# Ferroelastic switching in a layered-perovskite thin film

**DOI:** 10.1038/ncomms10636

**Published:** 2016-02-03

**Authors:** Chuanshou Wang, Xiaoxing Ke, Jianjun Wang, Renrong Liang, Zhenlin Luo, Yu Tian, Di Yi, Qintong Zhang, Jing Wang, Xiu-Feng Han, Gustaaf Van Tendeloo, Long-Qing Chen, Ce-Wen Nan, Ramamoorthy Ramesh, Jinxing Zhang

**Affiliations:** 1 Department of Physics, Beijing Normal University, 100875 Beijing, China; 2 EMAT (Electron Microscopy for Materials Science), University of Antwerp, Groenenborgerlaan 171, B-2020 Antwerpen, Belgium; 3 Institute of Microstructures and Properties of Advanced Materials, Beijing University of Technology, 100124 Beijing, China; 4 State Key Laboratory of New Ceramics and Fine Processing, School of Materials Science and Engineering, Tsinghua University, 100084 Beijing, China; 5 Tsinghua National Laboratory for Information Science and Technology, Institute of Microelectronics, Tsinghua University, 100084 Beijing, China; 6 National Synchrotron Radiation Laboratory and CAS Key Laboratory of Materials for Energy Conversion, University of Science and Technology of China, 230026 Hefei, China; 7 Department of Materials Science and Engineering, University of California, 94720 Berkeley, California, USA; 8 Beijing National Laboratory of Condensed Matter Physics, Institute of Physics, Chinese Academy of Science, 100190 Beijing, China; 9 Department of Materials Science and Engineering, The Pennsylvania State University, University Park, Pennsylvania, 16802 Pennsylvania, USA

## Abstract

A controllable ferroelastic switching in ferroelectric/multiferroic oxides is highly desirable due to the non-volatile strain and possible coupling between lattice and other order parameter in heterostructures. However, a substrate clamping usually inhibits their elastic deformation in thin films without micro/nano-patterned structure so that the integration of the non-volatile strain with thin film devices is challenging. Here, we report that reversible in-plane elastic switching with a non-volatile strain of approximately 0.4% can be achieved in layered-perovskite Bi_2_WO_6_ thin films, where the ferroelectric polarization rotates by 90° within four in-plane preferred orientations. Phase-field simulation indicates that the energy barrier of ferroelastic switching in orthorhombic Bi_2_WO_6_ film is ten times lower than the one in PbTiO_3_ films, revealing the origin of the switching with negligible substrate constraint. The reversible control of the in-plane strain in this layered-perovskite thin film demonstrates a new pathway to integrate mechanical deformation with nanoscale electronic and/or magnetoelectronic applications.

## Abstract (web summary)

Ferroelastic switching in thin films is typically restricted by constraints from the substrate or occurs around twin-like domains. Here, the authors show reversible and non-volatile ferroelastic switching avoiding substrate constraints in layered-perovskite Bi_2WO_6 epitaxial films.

## Graphical abstract

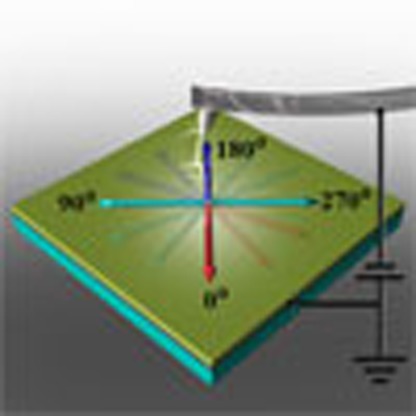

In ferroic (ferroelectric^1^,^2^, multiferroic^3^,^4^) materials and their heterostructures, a large reversible electric-field-driven elastic deformation can provide an effective pathway to achieve the coupling between lattice degree of freedom and other order such as spontaneous polarization, spin, orbital and so on^5^,^6^,^7^,^8^,^9^,^10^. This is crucial for designing sensing, data-storage and magnetoelectric devices with ultralow energy consumption^11^,^12^. In order to integrate these functionalities into applications, the pursuit of such an electric-field-induced elastic deformation in a thin film heterostructure, particularly a large ferroelastic strain controlled within a mono-domain structure, is highly desirable^13^,^14^,^15^. However, the ferroelastic switching (non-180° polar rotation) in traditional epitaxial ferroelectric or relaxor thin films suffers significant constraint from the substrate^16^,^17^, inhibiting the intrinsic non-volatile in-plane elastic strain and the mechanical coupling at hetero-interfaces^18^. The main origins of clamping are the elastic constraint of the vertical deformation in a thin film on a substrate geometry as well as the domain-wall pinning around the film/substrate interface^19^,^20^,^21^,^22^,^23^,^24^,^25^. One effective way to reduce the substrate clamping is micro-patterning of those oxide thin films using a method such as focused ion beam (FIB) etching to optimize the aspect ratio of the thin films^24^,^25^,^26^,^27^. This, however, usually requires a complicated process in nanoscale thin film devices^15^. Very recently, chemical doping or epitaxial misfit strain^28^,^29^,^30^ in tetragonal or rhombohedral ferroelectric thin films seems to be a possible solution to facilitate an in-plane ferroelastic switching. However, the polarization switching only exists around an array of twin-like domain or multi-domain structures, indicating that the elastic strain is still clamped and a controllable switching of a large-area mono-domain with pure elastic switching for practical applications is difficult^31^.

Under these circumstances, there is a strong impetus to explore new ferroelectric material systems, where the ferroelastic switching and non-volatile in-plane strain in a thin film heterostructure may not be restricted by the substrate. The elastic switching and strong substrate clamping in ferroelectric thin films with a projection of out-of-plane polarization is dependent on the crystal symmetry and geometry of the nanostructures with specific domains^16^,^24^. They can be described as:


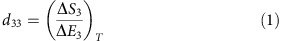



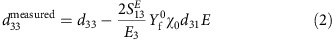


where *S*_3_ (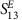) is the out-of-plane strain caused by the applied electric field *E*_3_ (the subscript 3 is defined as out-of-plane direction and the subscript 1 is defined as the in-plane direction.); *d*_33_ and 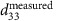 are the intrinsic piezoelectric coefficient and the effective piezoelectric coefficient, respectively; 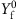 is the generalized Young's modulus of the film and *χ*_0_ is the distribution function of the stress^24^. It indicates that the out-of-plane and/or in-plane crystal deformation in a thin film structure is mostly restricted by the intrinsic *d*_31_ of the material. Considering a pure in-plane polarized thin film without any possible out-of-plane projection and switching (the intrinsic *d*_31_ is negligible), it will not give rise to out-of-plane deformation before and after the application of the out-of-plane or in-plane electric field. Polarization will only switch among the in-plane equivalent states so that the switching energy barrier or penalty from the change of volume will be quite small compared with the case in ferroelectric materials with the possibility of out-of-plane switching. Previous theoretical work has predicted that a pure in-plane ferroelectric polarization could be achieved in a ferroelectric thin film with an orthorhombic symmetry^32^,^33^. For traditional ferroelectric oxides (for example, BiFeO_3_ (BFO), PbTiO_3_ (PTO), BaTiO_3_ (BTO) and so on), the stabilization of the orthorhombic phase with a robust ferroelastic switching and control of the ferroelectric domains at room temperature is very challenging^28^,^29^,^30^,^32^,^33^. Alternatively, Aurivillius-layered perovskites are ferroelectric with an orthorhombic ground state^34^,^35^,^36^,^37^ and a high Curie temperature (ranging from 650 to 1,200 K). In these compounds, the primary ferroelectric polarization is along the [100] crystallographic direction due to the Bi displacement in the Bi_2_O_2_ fluorite-like sheet with respect to their octahedral^38^,^39^, supporting a 90° elastic domain wall in their (001) plane of those single crystals^40^,^41^. Therefore, the full in-plane polarization of 001-oriented epitaxial thin film heterostructures of Aurivillius oxides could provide a new framework for the in-plane ferroelastic switching.

In this work, we take ferroelectric Bi_2_WO_6_ (BWO), the simplest structure in the Aurivillius family^38^,^42^,^43^, as a model system to demonstrate an approach to obtain a large in-plane ferroelastic strain in 001-oriented epitaxial thin films. Scanning probe microscopy studies reveal that the ferroelectric polarization is fully constrained within the film plane without any out-of-plane projection, forming elastic 90° domain walls. A full in-plane polarization switching using planar large-area electrodes produces a reversible in-plane strain of approximately 0.4%, which has been confirmed by high-resolution (scanning) transmission electron microscopy (STEM) and X-ray diffraction. In combination with phase-field simulation, thickness-dependent and probe-based switching experiments provide further insights into the microscopic origin of the in-plane ferroelastic polar rotation with respect to fully clamped ferroelectric PbTiO_3_ thin films.

## Results

### Epitaxial growth of Bi_2_WO_6_ thin film

**Figure 1:**
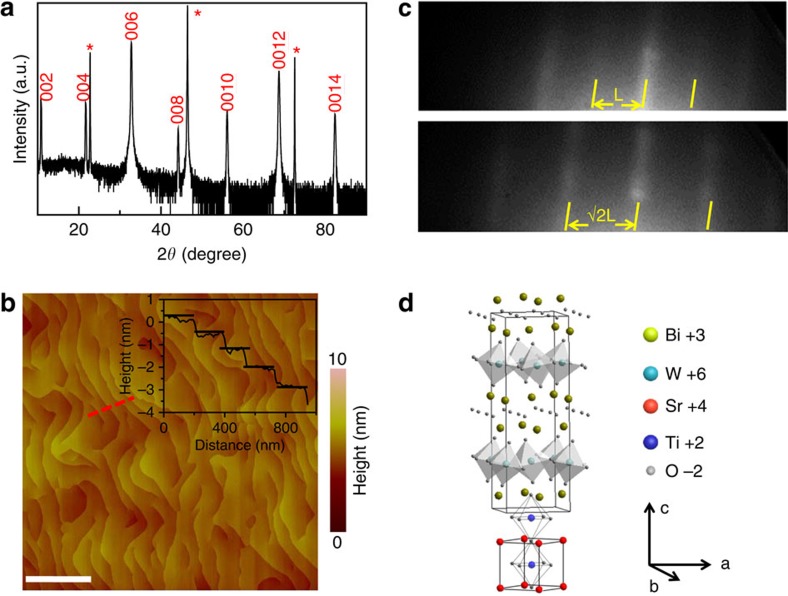
Crystallographic structure of the epitaxial BWO films. (**a**) The *θ*-2*θ* X-ray diffraction pattern indicates a typical 001-oriented epitaxial Aurivillius oxide thin film (*indicates that the peaks belong to the STO substrate). (**b**) Topography of the high-quality epitaxial BWO thin film. The inset shows an average step height with about 0.81 nm derived from the area indicated as the red dashed line. Scale bar, 1 μm. (**c**) RHEED patterns (indicated by the yellow lines) before and after the epitaxial growth of ferroelectric BWO (100; top panel) on STO (100; bottom panel). The distance between them is indicated as the yellow double-sided arrows. (**d**) Schematic diagram of the epitaxial relationship between the film and the substrate.

Epitaxial BWO thin films (approximately 200 nm) have been grown using laser molecular beam epitaxy on perovskite substrates such as SrTiO_3_ (STO) or LaAlO_3_ (LAO). The detailed growth conditions can be found in the Methods section or elsewhere^44^. Our *θ*-2*θ* X-ray diffraction analysis of the film on a STO substrate reveals a high-quality epitaxial structure with a set of 001-oriented diffraction peaks as shown in Fig. 1a. A low-magnification TEM study and energy dispersive X-ray spectroscopy analysis confirm that the chemical composition of the as-grown epitaxial BWO film (Supplementary Fig. 1) is very close to that of the desired BWO phase. High-resolution atomic force microscopy (AFM; Fig. 1b) shows the step-flow growth of the layered-perovskite with a step height of ∼0.81 nm (half a BWO unit cell, as seen in the inset), while in the bulk the lattice constants are a=5.437 Å, b=5.458 Å and c=16.430 Å, respectively^42^,^43^. According to the *in situ* reflection high-energy electron diffraction (RHEED) pattern in Fig. 1c, the distance of characteristic diffraction spots of the film is √2 times smaller than the one of the STO substrate, indicating that the in-plane lattice constant of the film is approximately √2 times larger than the one of the STO substrate (a=3.935 Å at growth temperature), which is ∼5.564 Å. This reveals a 45° in-plane rotation of the crystal orientation around the *c*-axis between film and substrate. This epitaxial relationship between BWO and the STO substrate is schematically illustrated in Fig. 1d. The detailed measurements for *θ*-2*θ* X-ray diffraction, AFM and STEM are given in the Methods section. The epitaxial growth of high-quality BWO films provides us with a fundamental model system of Aurivillius-layered compounds and allows us to explore their domain structures and ferroelastic switching mechanism, which is the central theme of this work.

### Structure of ferroelectric/ferroelastic domain

**Figure 2:**
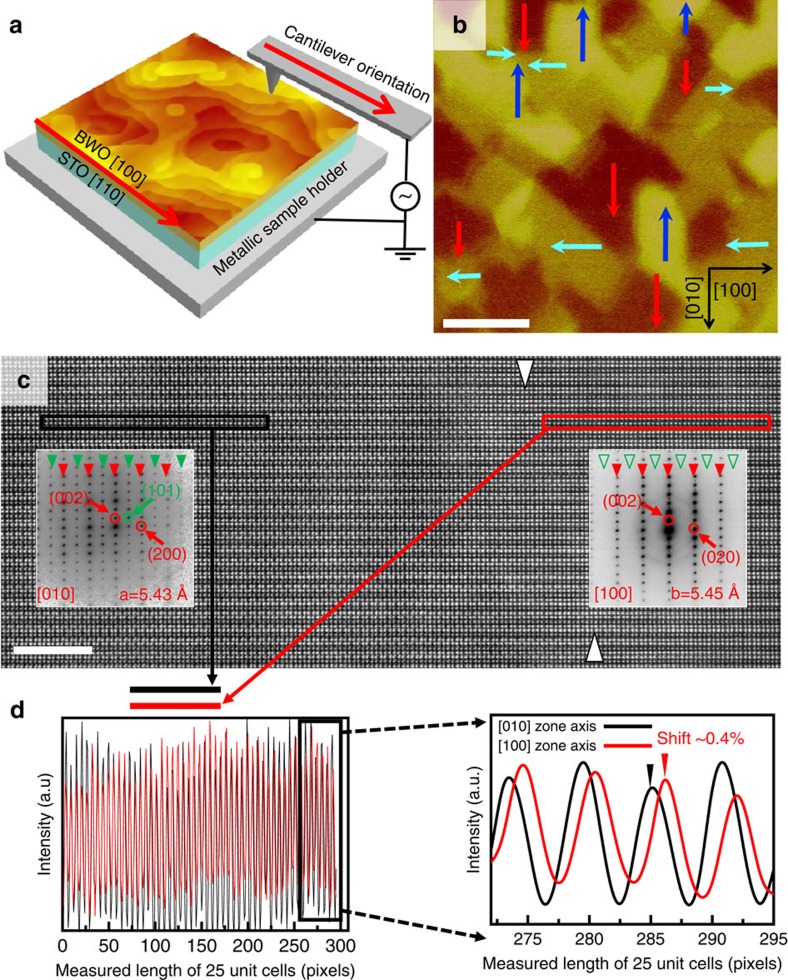
Observation of ferroelectric-ferroelastic domains. (**a**) A Schematic geometry of the PFM measurement on BWO film grown on STO substrate (an AFM image was inserted). (**b**) The corresponding in-plane PFM image shows the in-plane ferroelectric domain structure with four preferred in-plane polarization directions labelled by blue, red and cyan arrows, respectively (the crystallographic directions of BWO film are labelled by black arrows). Scale bar, 500 nm. (**c**) Atomic-resolution HAADF-STEM image reveals a ferroelastic domain boundary (indicated by two white arrows) between two ferroelectric domains (*a* and *b* domains viewed along [010] and [100] zone axis, respectively); the inserted electron diffraction patterns confirm the change in orientation. Scale bar, 5 nm. (**d**) Line scans of 25 unit cells measured at both sides of the ferroelastic domain wall showing a spontaneous strain. The two periods of the intensity modulation (indicated by the red and black colour corresponding to the frame colours in **c**, respectively) are aligned to the starting peaks of unit cells, and show a distinctive shift with regard to each other after 25 unit cells (zoomed-in shown in the inset), corresponding to a measured strain of approximately 0.4%.

The ferroelectric domain configuration of BWO thin films (approximately 200 nm) on STO substrates has been acquired using a scanning-probe-based technique as shown in the piezoresponse force microscopy (PFM) images in Fig. 2a,b. A schematic geometry of the PFM measurement on BWO film grown on STO substrate is illustrated in Fig. 2a. The polarization vectors are indicated by blue, red and cyan arrows in the in-plane domain image of Fig. 2b. A re-construction of the pure in-plane ferroelectric domain and polar vectors can be seen in Supplementary Fig. 2. More detailed PFM measurements can be seen in the Methods section. The study of the ferroelectric domain structure confirms that the ferroelectric polar directions of the BWO film is along the <110> direction of the perovskite substrate. In this 001-oriented orthorhombic ferroelectric oxide, there are four preferred in-plane polarization directions, indicating the presence of 90° ferroelastic domain walls in BWO thin films grown on various perovskite substrates such as LAO and STO, which can be seen in the in-plane PFM image in Supplementary Fig. 3. The BWO films grown on STO suffer in-plane tensile strain (see X-ray diffraction analysis in Supplementary Fig. 3). An epitaxial tensile strain (approximately 1.7% lattice mismatch between BWO and STO) can further stabilize the in-plane ferroelectric polarization^32^,^33^. Therefore, the microscopic structure of the ferroelastic domain walls of the epitaxial BWO/STO heterostructure has been analysed.

A cross-sectional high-angle annular dark-field STEM (HAADF-STEM) image in Fig. 2c shows a 90° domain wall of the epitaxial BWO film at atomic resolution. The domain wall is ill-defined, but clearly visible from the diffraction patterns on both sides, as demonstrated in the insets. Across the ferroelastic domain wall (indicated by two arrows), ferroelectric *a* and *b* domains (viewed along [010] and [100] crystallographic directions, respectively) of BWO are revealed at each side. Different lattice constants (5.43±0.01 Å versus 5.46±0.01 Å) and electron diffraction patterns are obtained along both zone axes as shown in the inset, where extra reflections of *(2n+1, 0, m)* viewed along [010] are indicated by solid green arrows, exampled by (101). On the other hand, these extra reflections are absent due to higher structural symmetry when viewed along the zone axes of [100], as indicated by hollow green arrows. The distinct differences in lattice constant between both domains are clearly evidenced by taking line scans over 25 unit cells from the [100] and [010] domains of BWO thin film (Fig. 2d) calibrated using internal reference of the STO substrate, indicating an approximately 0.4% elastic strain across the ferroelastic domain wall. Such an in-plane strain over a large area of the film has been further confirmed in reciprocal space mapping (RSM) as seen in Supplementary Fig. 4. The detailed measurements for the high-resolution STEM and RSM can be seen in the Methods section.

### Controllable ferroelastic switching

**Figure 3:**
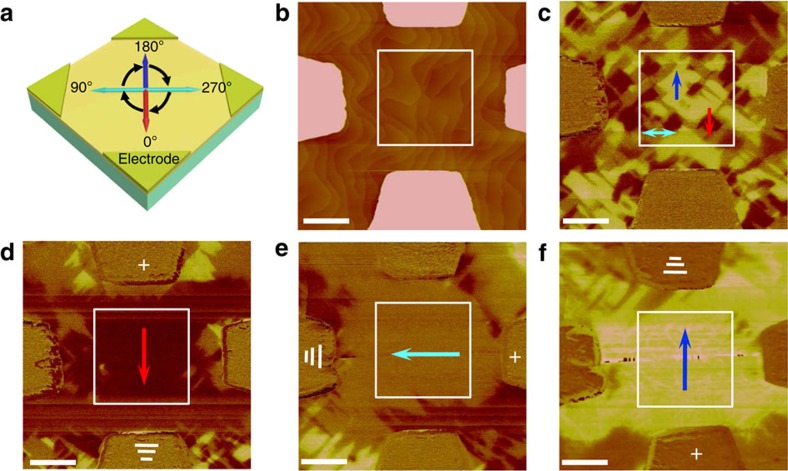
Deterministic control of ferroelastic mono-domain switching via planar electrodes. (**a**) Schematic presentation of domain ferroelastic switching with four in-plane electrodes (red, blue and cyan arrows indicate four polarization directions and the black arrows indicate the switching with 90° step). (**b**) AFM image of the BWO thin film directly grown on STO substrate with four in-plane electrodes. (**c**) The PFM image shows the as-grown ferroelectric domain structure before the application of the in-plane electric field. (**d**) The PFM image shows a mono-domain structure when an in-plane electric field is applied on BWO. (**e**,**f**) Ferroelectric mono-domains with a polarization sequentially rotated by 90° (ferroelastic switching) when the in-plane electric field is further applied. Scale bar, 1 μm (**b**–**f**).

In order to demonstrate an in-plane ferroelastic switching with 90° polarization rotation in the layered-perovskite BWO thin films with a negligible constraint from the substrate (illustrated schematically in Fig. 3a), Au-electrode patterns were fabricated on top of the BWO/STO heterostructure (film thickness approximately 200 nm) as seen in the AFM image in Fig. 3b. *In situ* in-plane PFM studies (Fig. 3c–f) before and after the application of the electric field (about 50–200 kV cm^−1^) provide direct evidence that the ferroelastic domains are switchable without in-plane constraints. Figure 3c shows the as-grown multi-domain structure with four preferred polarizations before the application of the electric field. When an electrical bias was applied on the top-viewed electrode, the as-grown multiple-domain structures started to switch and were eventually erased (up to 200 kV cm^−1^) within the electrodes. A ferroelectric mono-domain appeared with its polarization along the direction of the applied electric field (Fig. 3d). The emerging large-area mono-domain can be sequentially switched by 90° as a function of the electric field as shown in Fig. 3e,f. The microstructure and the correlated elastic strain before and after the ferroelastic domain switching have been investigated using dark-field TEM and HAADF-STEM. The switched mono-domain structure remains stable after at least 2 weeks across the orthorhombic crystal as shown in Supplementary Fig. 5, indicating that an electric-field-driven in-plane non-volatile elastic strain can be achieved in the whole region beneath the surface of the ferroelectric thin film.

The controllable ferroelastic strain of approximately 0.4% has been further demonstrated on the electrically switched *a* and *b* domains of BWO film using (S)TEM, as shown in Supplementary Fig. 6. Supplementary Figure 6a and e are the PFM images of the ferroelectric domains after the electrical switching. Both of two lamellae are then vertically extracted (along and perpendicular to the directions of the electric field in Supplementary Fig. 6a,e) from the switched regions for (S)TEM investigations, respectively. Dark-field TEM (Supplementary Fig. 6b,f) reveals that a single region of one large domain is successfully patterned as characterized by the uniform contrast in-between the gold contacts. Diffraction patterns from each lamella are shown in Supplementary Fig. 6c,g, indicating the viewing directions of [100] and [010], that is, the electrically polarized directions of [010] (*b* domain) and [100] (*a* domain), respectively. Supplementary Figure 6d and h are the corresponding high-resolution HAADF-STEM images of two switched *b* and *a* domains. Line scans of 25 unit cells from each domain (calibrated using internal reference of STO substrate) show a controllable strain of approximately 0.4% between switched pure *a* and *b* domains (Supplementary Fig. 6i). It is noted that the strain value is not obtained directly from the same region before and after electrical switching, while the diffraction patterns and (S)TEM results along [100] and [010] directions on any switched region calibrated using the STO substrate help provide the circumstantial evidence of this electrically controllable strain of ∼0.4% on polarized domains. The detailed methods of the fabrication of the micro-sized electrodes, electric field control process and the *ex situ* TEM sample preparation can be found in Methods section.

### Origin of the elastic switching with negligible constraint

**Figure 4:**
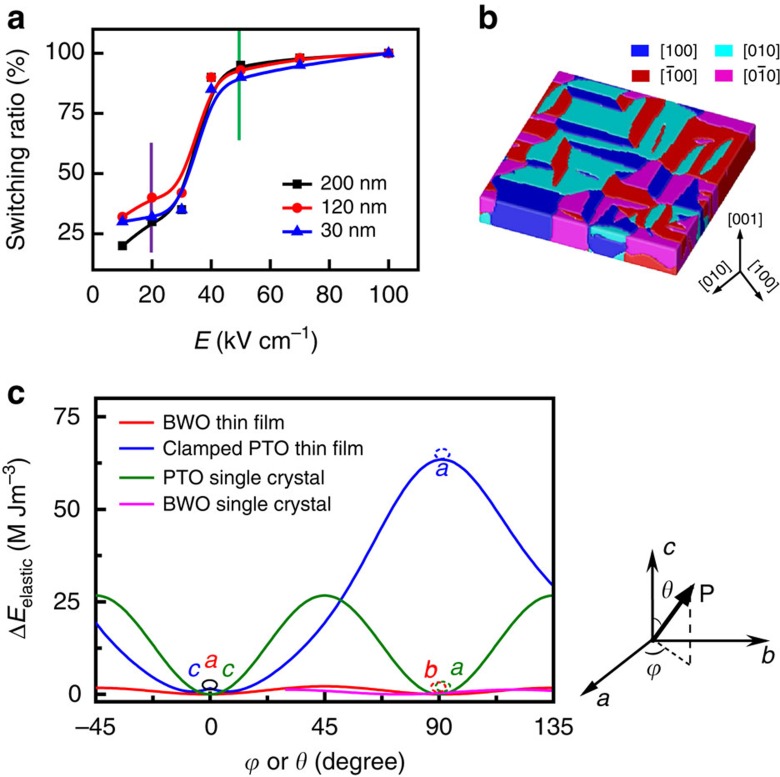
Ferroelastic switching mechanism. (**a**) Ferroelastic switching areal ratio as a function of planar electric fields in BWO thin films with thicknesses of 30, 120 and 200 nm, respectively. It shows that the electric fields for elastic switching maintain in the BWO thin films with various thicknesses which have a similar threshold switching field about 20 kV cm^−1^ (indicated as purple line) and a full switching field about 50 kV cm^−1^ (indicated as green line). (**b**) Simulated domain structure of the 001-oriented BWO crystal with four preferred polarization directions indicated by blue, red, cyan and pink colour, respectively. The crystallographic directions of BWO are indicated as the black arrows, respectively. (**c**) Phase-field studies of the energy potential curves between PTO and BWO during the electric-field-driven ferroelastic domain switching in bulk and thin film, respectively; the energy potential for a ferroelastic switching in the BWO thin film on STO is approximately 15 times lower than the one for a ferroelastic switching in a fully clamped PTO thin film on STO, indicating that the substrate constraint on ferroelastic switching is negligible in BWO epitaxial film.

To further elucidate the mechanism of the ferroelastic switching and in-plane non-volatile strain in BWO thin films, we studied the thickness-dependence of the switching field and used phase-field modelling to understand the electric-field-induced elastic mono-domain switching. First, the magnitude of substrate clamping and the consequent ferroelastic switching usually depends on the thickness of epitaxial oxide thin films^45^,^46^. For the BWO film with pure in-plane polarization, we studied the switching areal ratio as a function of electric field using planar electrodes in BWO heterostructures with thicknesses of 30, 120 and 200 nm, where we can see that domain size is essentially constant as a function of thickness (Supplementary Fig. 7). In Fig. 4a, we clearly observed that there is similar full switching field (approximately 50 kV cm^−1^) for all these thicknesses, which means that the in-plane elastic switching is independent of the epitaxial strain release due to the increase of thickness. Unlike the domain switching in out-of-plane switching ferroelectrics, this result demonstrates that the in-plane polar rotation and free elastic switching is attributed to the intrinsic orthorhombic symmetry in this layered-perovskite.

We calculated the potential energy barriers (Fig. 4c) resulting from the ferroelastic switching in PTO epitaxial thin films on STO, PTO single crystals, BWO epitaxial thin films on STO and BWO single crystals, respectively. The detailed method for simulation is given in the Supplementary Note 1. As shown in Fig. 4b, there are four preferred ferroelectric polarizations along the <100> directions within the (001) plane of BWO, consistent with our experimental observations. In a fully clamped 001-oriented tetragonal PTO thin film, a 90° ferroelectric domain switching (transition between *a* and *c* domain) driven by an electric field is energetically unfavourable (without an energy minimum around 90° in the blue curve) and only 180° switching is possible from a thermodynamic point of view^23^. In a PTO single crystal without substrate clamping, we observe two energy minima during the ferroelastic switching (green curve), indicating two elastic domain states with a decreased energy potential of about 27 MJ m^−3^ (corresponding to an electric field of approximately 380 kV cm^−1^ for the elastic domain switching). In an orthorhombic BWO, double-well energy potentials are revealed, where the energy potential required for 90° switching within the (001) plane is about 5 MJ m^−3^ for both the epitaxial film and single crystal (approximately 15 times lower than the one of a fully clamped PTO thin film) as indicated by the red and pink curves. This energy corresponds to an electric field of approximately 50 kV cm^−1^, which is consistent with our experimental observations in the electric-field-driven elastic switching of the BWO thin film.

In order to compare the in-plane elastic switching in BWO and a fully clamped PTO thin film using planar electrodes, we used a PTO film on KaTiO_3_ (KTO) substrate^47^,^48^ to analyse the switching field and the switching energy when the polarization rotation occurs from twin-like to pure *a* or pure *b* domain (two variants of the in-plane polarization), where ferroelectric polarization is simulated to be fully in the film plane due to the epitaxial strain in PTO/KTO heterostructure. The detailed method and results for simulation are given in the Supplementary Note 1 and Supplementary Fig. 8. As shown in Supplementary Fig. 8a,b, the electric field for in-plane elastic switching from multi-domain to mono-domain in fully clamped PTO is above 4,000 kV cm^−1^, which is over one order of magnitude larger than the one of BWO film. Such a large in-plane electric field for the elastic switching in a PTO film due to the substrate clamping is technically impractical^13^,^14^,^15^. The phase-field simulation further indicates that the substrate constraint on the ferroelastic switching is negligible in this intrinsic orthorhombic BWO ferroelectric thin film with a pure in-plane polarization in contrast to other ferroelectric thin films with tetragonal or rhombohedral ground states.

### Nanoscale control of domain features and elastic switching

**Figure 5:**
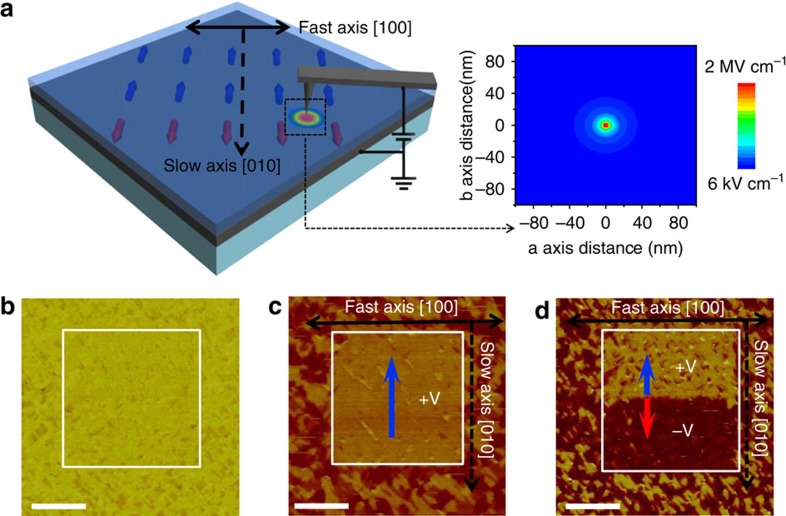
Nanoscale control of multiple ferroelectric/ferroelastic domain features. (**a**) Schematic geometries of the in-plane ferroelastic domain structure of BWO film grown on STO substrate with SRO bottom electrode controlled by an out-of-plane electric field applied on the AFM tip and the distribution of the in-plane electric field underneath the tip. The blue and red arrows label two polar vectors perpendicular to the fast-scanning axis of the BWO [100] crystallographic direction (indicated by the black line with double-sided arrows). (**b**) The out-of-plane PFM image shows no out-of-plane projections of the ferroelectric polarization before and after the application of the probe bias. (**c**) The in-plane PFM image acquired after the probe-bias switching with a +10 V bias applied on the scanning tip, where the polarization direction of the switched domain is anti-parallel to the slow-scanning direction as illustrated by the blue arrow. (**d**) In-plane PFM image acquired after the probe-bias switching with a +10 and −10 V bias applied alternatively on the scanning tip, where the polarization directions of the switched domains are anti-parallel and parallel to the slow-scanning direction as illustrated by the red and blue arrows. Scale bar, 1 μm (**b**–**d**).

With the full understanding of the in-plane clamping-free elastic switching in BWO thin films, nanoscale manipulation of this elastic strain and mono-domain structure help further reveal the mechanism of the ferroelastic switching dynamics without the assistance of twin-like nanodomains. Scanning-probe-based technique is a powerful tool to generate a local electric field and control the ferroelectric polarization within a size of approximately 20 nm (refs 49, 50). The nucleated mono-domain may depend on the tip-scanning size and direction, which is not restricted by the large voltage and geometry in planar electrodes. As illustrated schematically in Fig. 5a, a conductive epitaxial SrRuO_3_ (SRO) was used as a bottom contact in the BWO/SRO/STO heterostructure during the nanoscale probe bias control, where a simulated distribution of the in-plane electric field beneath the nanoscale metallic AFM tip is also shown. For all the probe bias poling in the BWO/SRO heterostructures, out-of-plane domain switching is absent as confirmed by the out-of-plane domain image before and after the application of the electric field (Fig. 5b), indicating the polarization is fully in the film plane. This behaviour can be also confirmed by the dielectric measurement as a function of electric field (Supplementary Fig. 9g). In an approximately 200-nm thick BWO thin film, the in-plane ferroelectric polarization was totally switched under the application of a probe bias of approximately +10 V as shown in Fig. 5c. The switched in-plane polarization can be determined by the slow-scanning direction of the AFM cantilever and the probe bias voltage. As seen in Fig. 5c and Supplementary Fig. 10, the final in-plane polar vector (blue arrow) of BWO is always anti-parallel to the slow-scanning direction (dark dash arrow) when a positive bias is applied on the AFM tip. However, when a negative bias is applied on the AFM tip, the local field with opposite directions is reversed, resulting in a final polarization parallel to the slow-scanning direction (the red arrow in Fig. 5d). The detailed control of domain switching with scanning-probe-bias is given in the Supplementary Note 2.

**Figure 6:**
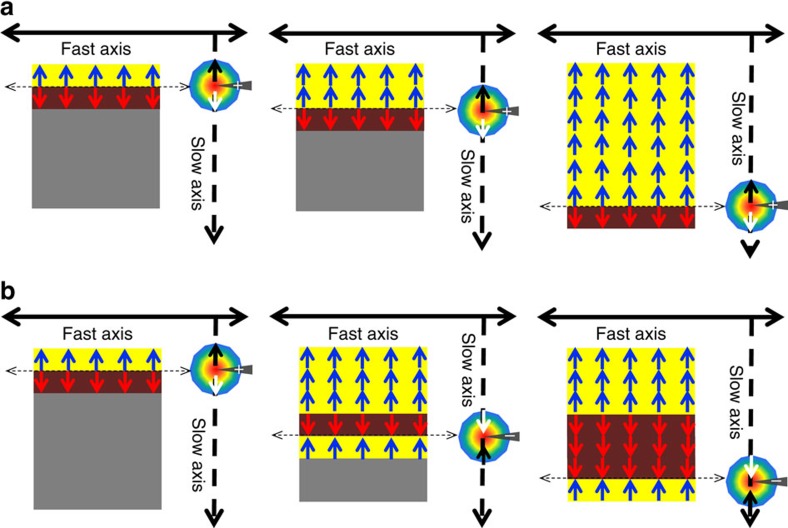
Scanning-probe switching dynamics at nanoscale. (**a**) Schematics of the in-plane polarization switching process under the application of a scanning-direction-dependent anisotropic electric field. (**b**) Schematics of the in-plane polarization switching using an anisotropic electric field with positive and negative bias. The [100] and [010] crystallographic directions of BWO are indicated by the black line and dashed line with double-sided arrows, respectively.

The dynamics of this probe-bias-induced pure in-plane switching are shown in Fig. 6. During the probe switching, the scanning-direction-dependent anisotropic in-plane component of a positive local bias applied on the AFM tip interacts with the in-plane polarization of BWO. Therefore, the in-plane polar vectors of BWO will be switched row-by-row by the local bias with opposite directions (dark and white arrows around the tip). In this way, polarization switching from a multi-domain state to a mono-domain state occurs, accompanying with the final polar vectors anti-parallel to the slow-scanning direction as seen in Fig. 6a. Consequently, an alternative positive and negative probe bias on the AFM tip will give rise to an opposite polarization (blue and red arrows) in the pure in-plane domain structure as shown in Fig. 6b. The ferroelastic switching of the BWO films with different thickness from 30 to 200 nm are also shown in Supplementary Fig. 11. Different with the strain-driven twin-like domain switching in tetragonal or rhombohedral ferroelectric thin films, the nanoscale manipulation of the in-plane ferroelastic switching dynamics in this intrinsic orthorhombic system is independent with the epitaxial strain. BWO films with −1.3% compressive strain show similar domain formation and switching behaviour as the one on STO substrate (Supplementary Fig. 12). This demonstrates that the switched mono-domain is totally decoupled with each other, which provides us a crucial pathway for the future exploration of domain-wall engineering and various topological structures^51^,^52^,^53^ in ferroelectric materials.

**Figure 7:**
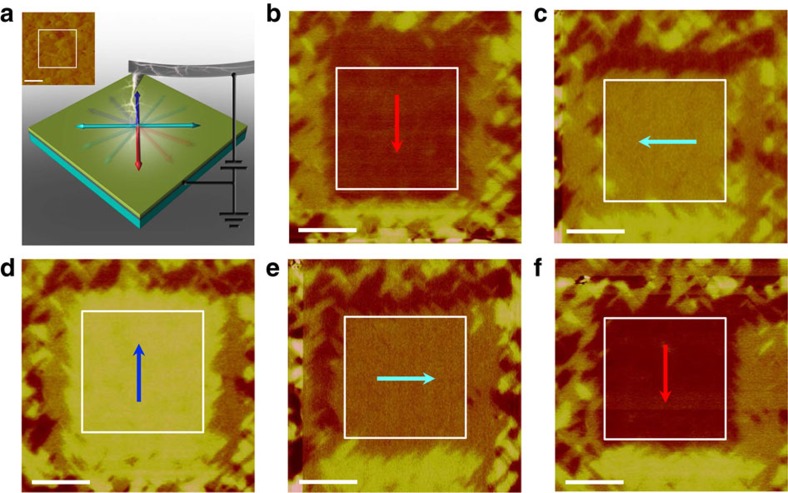
A complete 90° ferroelastic switching sequence. (**a**) The schematic of the ferroelastic switching with scanning-probe-bias of −15 V in the BWO thin film (approximately 120 nm) with SRO bottom electrode grown on STO substrate. The inset is the topography of the BWO thin film (the region in the white box is the switched area). Scale bar, 1 μm. (**b**) The PFM image of the downward-polarized mono-domain configuration as indicated by the red arrow. The controllable 90° ferroelastic switching can be achieved from a downward polarization to left polarization (**c**) as indicated by the cyan arrow, from left polarization to upward polarization (**d**) as indicated by the blue arrow, from upward polarization to right polarization (**e**) as indicated by the cyan arrow and finally, the mono-domain configuration can be switched back to the initial downward state for a complete sequence (**f**) as indicated by the red arrow. Scale bar, 1 μm (**b**–**f**).

Furthermore, a complete 90°ferroelastic switching sequence can be also reversibly controlled using the scanning-probe-based technique as seen in Fig. 7. This full in-plane rotation of polarization and a controllable ferroelastic mono-domain switching in thin film heterostructures without the assistance of twin-like nanodomains demonstrate a key step towards the integration of the ferroelasticity with a thin film geometry.

## Discussion

To conclude, we use epitaxial layered-perovskite BWO to demonstrate a ferroelastic switching with a reversible non-volatile control of mono-domain in an orthorhombic heterostructure, which is absent in ferroelectrics with rhombohedral or tetragonal structure due to their strong substrate clamping and pinning of domain-wall motion. In combination with phase-field simulation, manipulation of the elastic mono-domain switching can be achieved by a planar electrode and a scanning-probe-based technique, indicating that this material can be a good model system for ferroelastic domain engineering. The reversible control of nanoscale ferroelectric/elastic switching at room temperature with a significant in-plane non-volatile strain in a thin film heterostructure also provides us with a promising framework to study the coupling of the lattice degree of freedom with other order in future micro/nano applications.

## Methods

### Thin film growth

BWO and BWO/SRO heterostructures were fabricated using laser molecular beam epitaxy (248 nm excimer) with *in situ* monitoring of RHEED during the growth. Atomically smooth substrates (001-oriented STO and LAO) were prepared by a combined HF-etching/anneal treatment. Stoichiometric SRO and BWO (5% excess of Bi_2_O_3_ in order to compensate the volatile Bi at high growth temperature) targets were ablated at a laser energy density of approximately 1 J cm^−2^ and a repetition rate of 1 Hz for the growth of SRO (thickness approximately 10 nm) and BWO (thickness up to 200 nm), respectively. During growth of SRO, the substrate temperature was maintained at 700 °C at an oxygen environment of 100 mtorr. For the growth of BWO, the substrate temperature was 720 °C at an oxygen pressure of 100 mtorr. Afterwards, the films were cooled to room temperature at 0.1 atm of oxygen with a cooling rate of 5 °C min^−1^. The Au electrodes (approximately 50-nm thick) with micro-patterns were fabricated by magnetron sputtering assisted by electron beam lithography (JEOL JBX6300FS).

### Characterizations of crystal and domain structures

*X-ray diffraction*. X-ray *θ*-2*θ* scans were obtained by high-resolution X-ray diffraction (Lab XRD-6000, SHIMADZU). The detailed crystal structures was analysed by a four-circle film diffractometer with a Ge (220) × 2 incident-beam monochromator and stripe detector (Rigaku SmartLab Film Version, Cu-*Ka* radiation). For the strain values extracted from the rocking curve, the intensity distribution of diffraction spots could be described base on the kinetics of X-ray diffraction as:


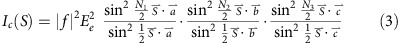


The profile of diffraction spots of small crystallite depends mainly on the geometry size of the crystallite. From this expression, it could be deduced that all the Bragg spots from one crystallite have almost the same rocking curve profiles in the reciprocal space, including the same FWHM value. In our sample, there are two kinds of crystalline domains: *a* and *b*. It is assumed that these two kinds of domains have the same average domain size along [100] direction. Thus the diffraction spots from *a* or *b* domain in the reciprocal space, such as (0 0 18), (2 0 18), (−2 0 18), should possess the same FWHM value in [100] direction. On the other hand, since the in-plane lattice parameters of BWO unit cell a and b are not the same, the diffraction spots at h0l(h≠0) from *a* and *b* domains should split along [100] direction, and the splitting interval stands for difference between lattice parameter a and b. In the case of BWO (2 0 18) or BWO (−2 0 18) RSM, due to the overlap of diffraction spots from *a* and *b* domains, the splitting interval is obtained indirectly by subtraction the size-caused width from the FWHM. The final value is about 0.0037 rlu of STO. The BWO (2 0 18) or BWO (−2 0 18) diffractions spots located around (1 1 4.285) in the reciprocal lattice units of STO. Therefore, the difference between lattice parameters a and b of BWO can be extracted by this method.

*FIB milling of the TEM samples*. The TEM samples (before and after the application of electric field) were prepared using FIB (FEI Helios NanoLab DualBeam system). Lamellae were prepared following the <110> direction of the STO substrate. A final cleaning of the sample surface was performed using 2 keV Ga^+^ ions with a small beam current to reduce amorphous layer on the lamellae. The thickness of the as-prepared lamellae is approximately 50–80 nm.

*TEM imaging conditions*. The high-resolution HAADF-STEM image was acquired using a FEI Titan Cube 60–300 microscope fitted with an aberration corrector for the probe-forming lens, operated at 300 kV. The STEM convergence semi-angle was approximately 21.4 mrad, providing a probe size of approximately 1.0 Å at 300 kV. Diffraction patterns and dark-field TEM images were acquired on a FEI Tecnai G2 microscope operated at 200 kV. Measured lattice constants of a and b of the as-grown thin film from HAADF-STEM images are a=5.43±0.01 Å and b=5.46±0.01 Å (error bar represents the s.d., which is calculated from 10 measurements from different areas using 2 samples).

*AFM and PFM*. The AFM measurements were carried out on tapping mode with ultra-sharp Si tips. The PFM measurements were carried out on a Bruker Multimode 8 AFM with commercially available TiPt-coated Si tips (Mikro Masch) with a tip curvature radius of <30 nm. The typical tip-scanning velocity was 2 μ ms^−1^. The amplitude and frequency of the AC input were 1.5 V_pp_ and 22 kHz, respectively. High-resolution PFM images were acquired on a wide array of samples. The polarization vectors have been re-constructed based on the domain images obtained from the cantilever scanning parallel and perpendicular to the BWO [100] axis, respectively.

## Additional information

**How to cite this article:** Wang, C. *et al.* Ferroelastic switching in a layered-perovskite thin film. *Nat. Commun.* 7:10636 doi: 10.1038/ncomms10636 (2016).

## Supplementary Material

Supplementary Information Supplementary Figures 1-12, Supplementary Notes 1-2 and Supplementary References
