# Supplementary material for: Ferroelastic switching in a layered-perovskite thin film
Source: Nat Commun. 2016 Feb 3;7:10636. doi: 10.1038/ncomms10636 (PMC4743001; doi:10.1038/ncomms10636)
Supplement: Supplementary Information — Supplementary Figures 1-12, Supplementary Notes 1-2 and Supplementary References [file ncomms10636-s1.pdf]

Supplementary Figures:

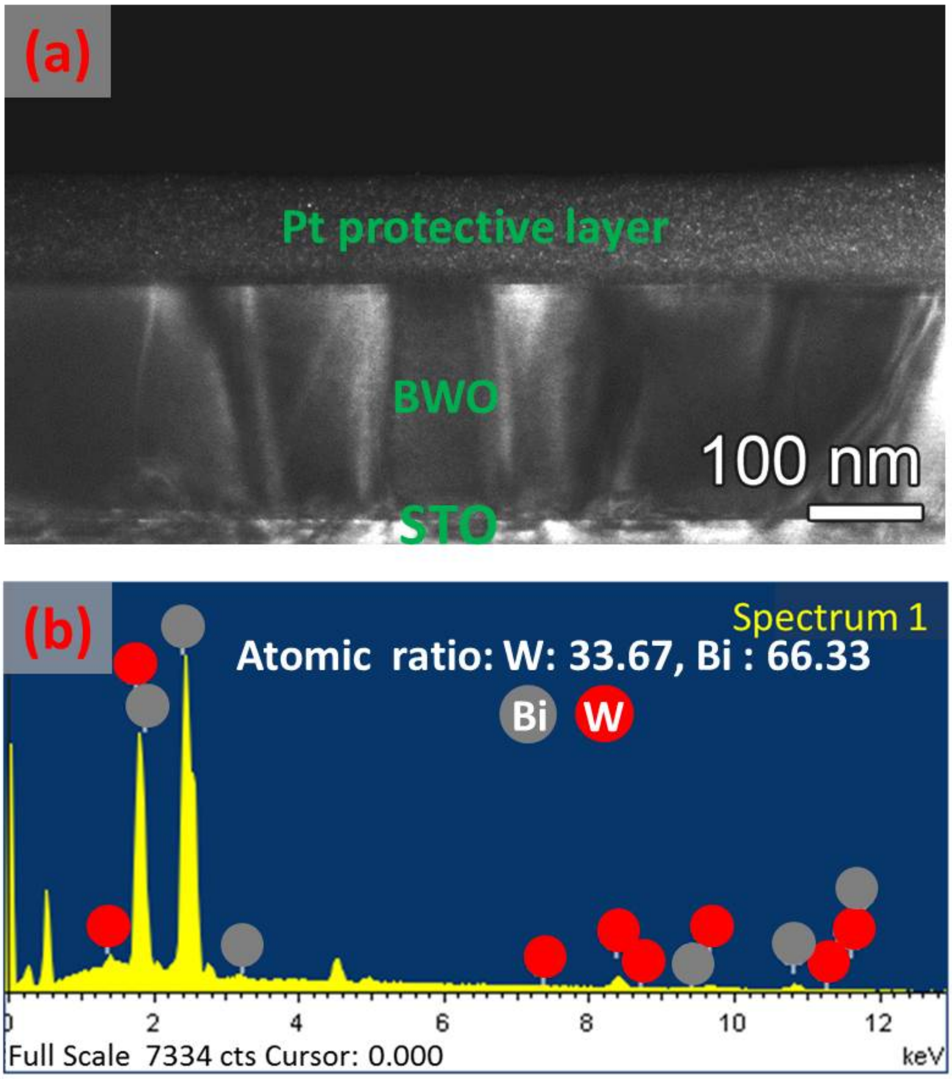

Supplementary Figure 1 | Cross-sectional morphology and Chemical composition. (a) A low-magnification dark-field TEM image shows the cross-sectional morphology of the BWO thin film with a thickness of ~200 nm. Scale bar, 100 nm. **(b)** An EDX analysis identifies the chemical molar ratio of Bi : W is about 2:1.

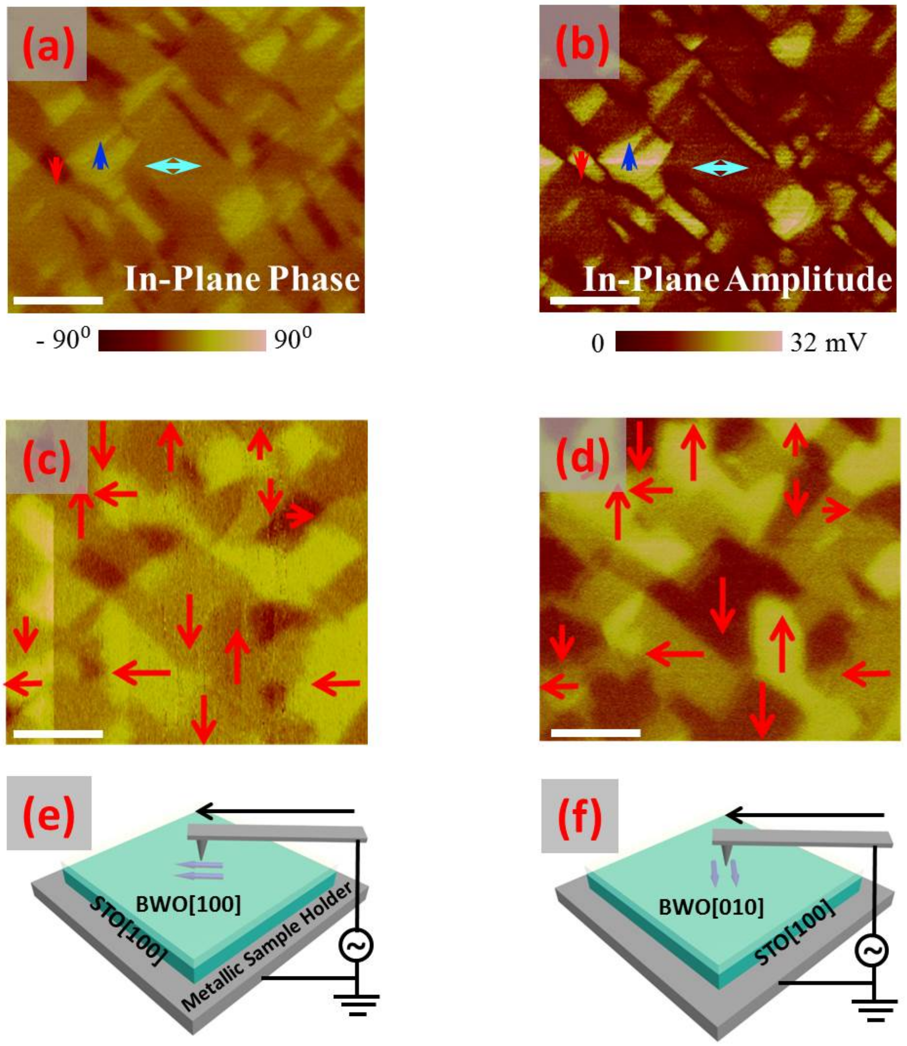

Supplementary Figure 2 | Re-construction of the pure in-plane ferroelectric domain. **(a)** The in-plane phase image of BWO film grown on (001) STO substrate with bottom electrode (the in-plane polarization directions labeled by blue, red and cyan arrows, respectively). **(b)** The corresponding in-plane amplitude image at the same area of the BWO film on STO substrate with bottom electrode. **(c)** The in-plane PFM image (AFM cantilever scanned along BWO [100] direction) without bottom electrode. **(d)** The in-plane PFM image without the bottom electrode (AFM cantilever scanned along BWO [010] at the same location as **(c)** shows the re-constructed in-plane polar vectors (red arrows). Scale bar, 500 nm **(a-d).** **(e)** and **(f)** schematic illustrations of the re-construction of ferroelectric polar vectors (blue arrows) in the PFM measurements.

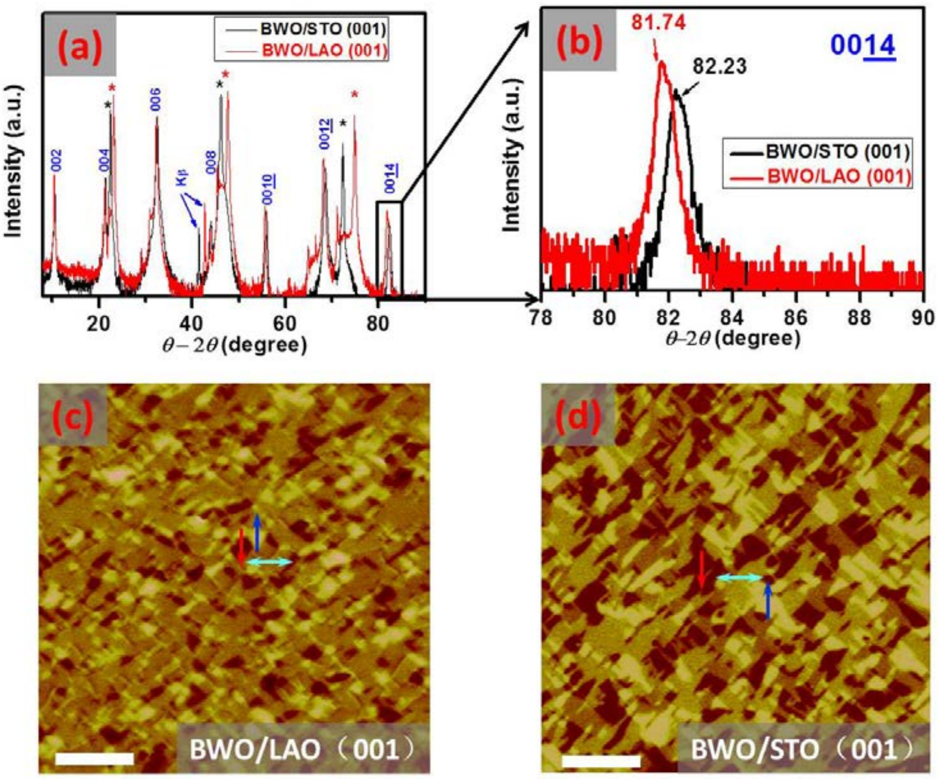

Supplementary Figure 3 | BWO films grown on different substrates. (a) The XRD patterns (θ-2θ scans) of BWO/LAO (001) and BWO/STO (001) films show the same crystallographic orientation of the high-quality epitaxial BWO thin films. **(b)** The zoom-in curves around the (0 0 $\underline{14}$) reflection of BWO thin films grown on LAO and STO substrates show a about 0.5% difference of out-of-plane lattice parameters of BWO thin films. The in-plane ferroelectric polar vectors of BWO thin films directly grown on LAO **(c)** and STO substrates **(d)** are indicated with the red, blue and cyan arrows. Sacle bar, 1 µm**(c-d)**.

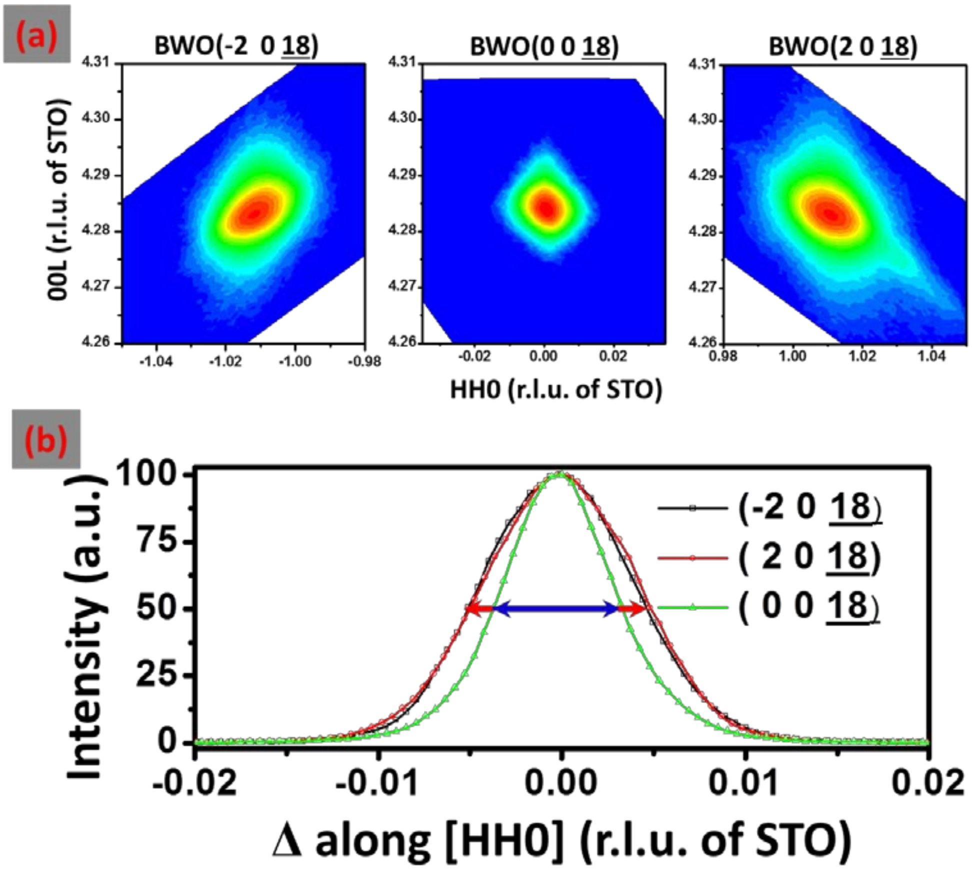

Supplementary Figure 4 | RSM of BWO thin film grown on STO. (a) Reflections around (-2 0 18), (0 0 18) and (2 0 18) of the BWO thin film. **(b)** The in-plane strain value can be estimated up to 0.4% from the full width at half maximum of the rocking curves crossing the diffraction spots in [HH0] direction.

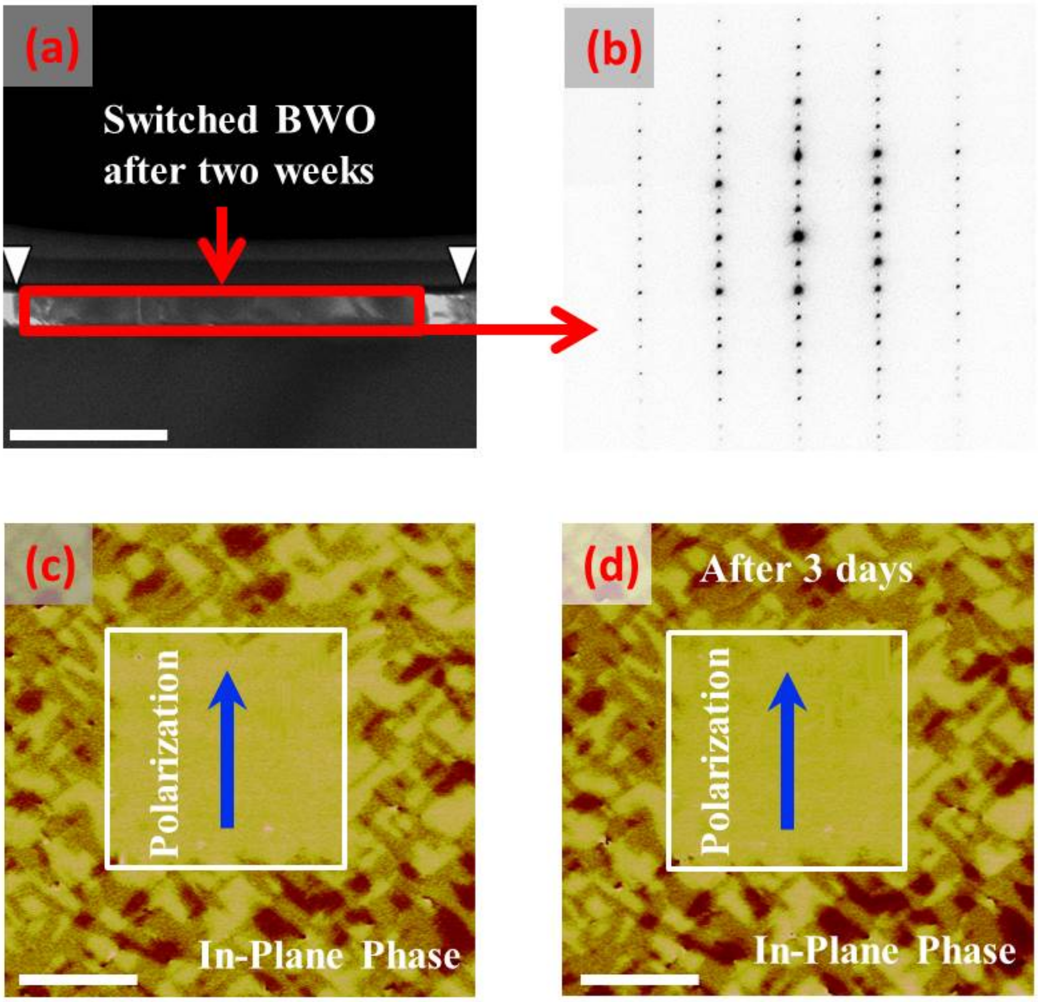

Supplementary Figure 5 | Non-volatile ferroelastic switching in BWO thin film. A cross-sectional dark-field TEM image **(a)** and the corresponding electron diffraction pattern **(b)** along [100] zone axes after the non-volatile ferroelastic switching in the area labeled in the red box (The switched mono-domain structure remains stable after at least two weeks). The white arrows in **(a)** indicate the positions of Au electrodes on BWO film directly grown on STO substrate. The PFM image **(c)** of the switched mono-domain structure of BWO film grown on STO substrate with SRO bottom electrode and the PFM image **(d)** measured at the same area after 3 days. Scale bar, 1μm **(a)** and **(c-d).**

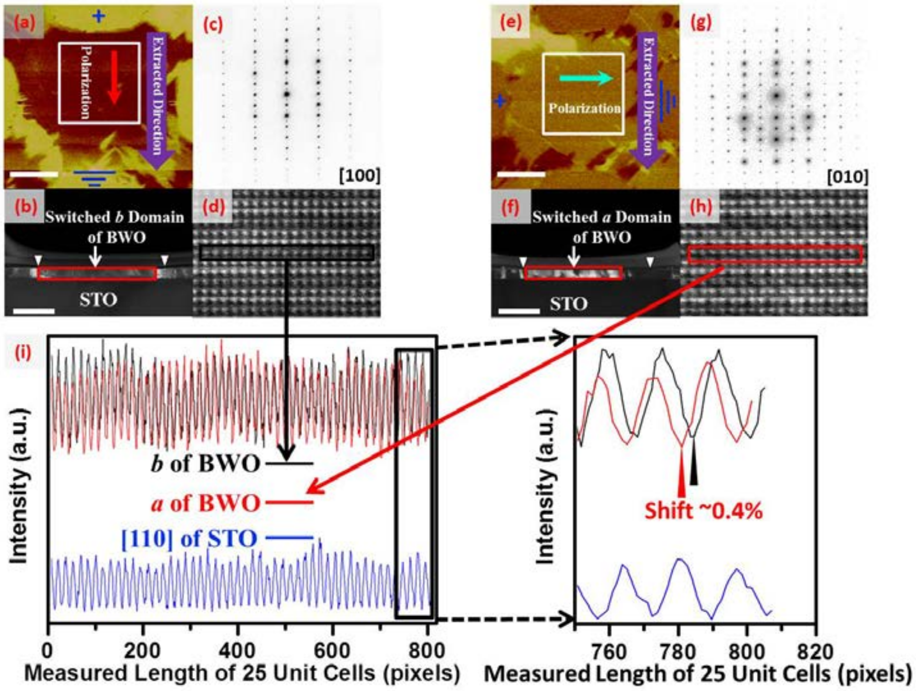

Supplementary Figure 6 | A controllable ferroelastic strain of approximately 0.4%. (a), (b), (c) and (d) are the PFM image (from samples without SRO bottom electrode), low-magnification dark-field TEM, the corresponding electron diffraction pattern and the atomic-resolution HAADF-STEM image of the BWO film with the micro-patterned structure, respectively. The observed region in the BWO film has been electrically switched along *b* axis (viewed along [100] crystallographic direction). **(e) (f), (g) and (h),** are the PFM image, low-magnification dark-field TEM, the corresponding electron diffraction pattern and the atomic-resolution HAADF-STEM image of the BWO film with the micro-patterned structure, respectively. The observed region in the BWO film has been electrically switched along *a* axis (viewed along [010] crystallographic direction). **(i)** Line scans of 25 unit cells measured between switched *a* and *b* domains (calibrated using internal reference of the STO substrate) circumstantially reveals that the controllable strain of approximately 0.4% can be achieved in a single region. Two periods of the intensity modulation are extracted from both domains, aligned to the starting peaks of each spectrum, and reveal a difference of approximately 0.4% after 25 unit cells. Sacle bar, 1 μm **(a-b)** and **(e-f)**.

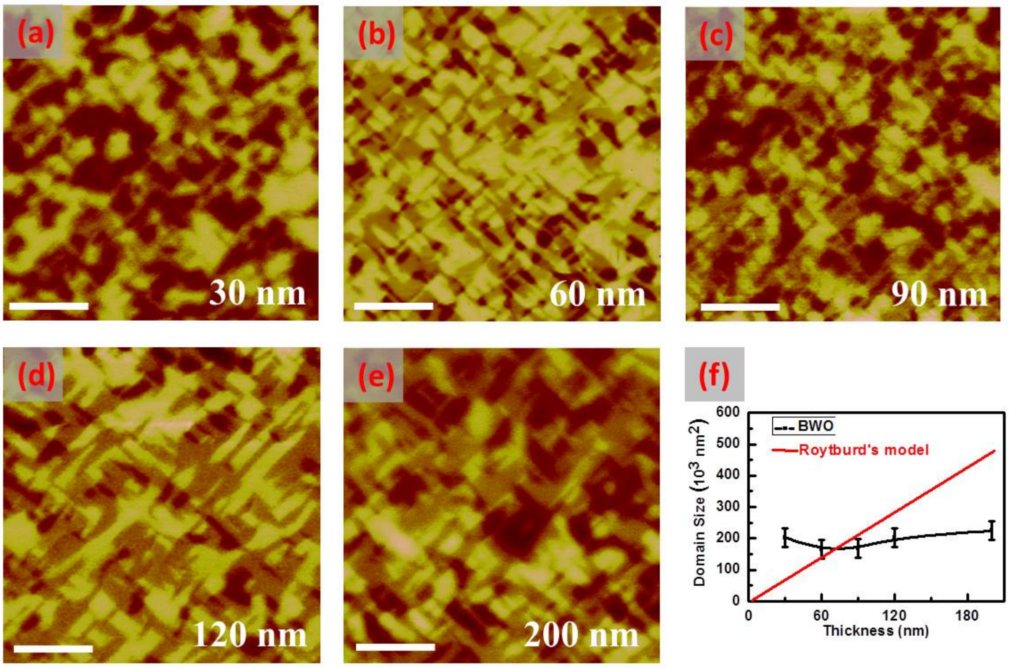

Supplementary Figure 7 | Evolution of BWO domain structure as a function of film thickness. The PFM images of the thin films directly grown on STO substrates with a thickness of **(a)** 30 nm, **(b)** 60 nm, **(c)** 90 nm, **(d)** 120 and **(e)** 200 nm, respectively. Sacle bar, 1 μm **(a-e)**. **(f),** the evolution of domain size of BWO with different thickness (the black line) and the comparison of the thickness-dependent domain size in other strain-induced ferroelastic domains (the red line) from Supplementary Ref.1.

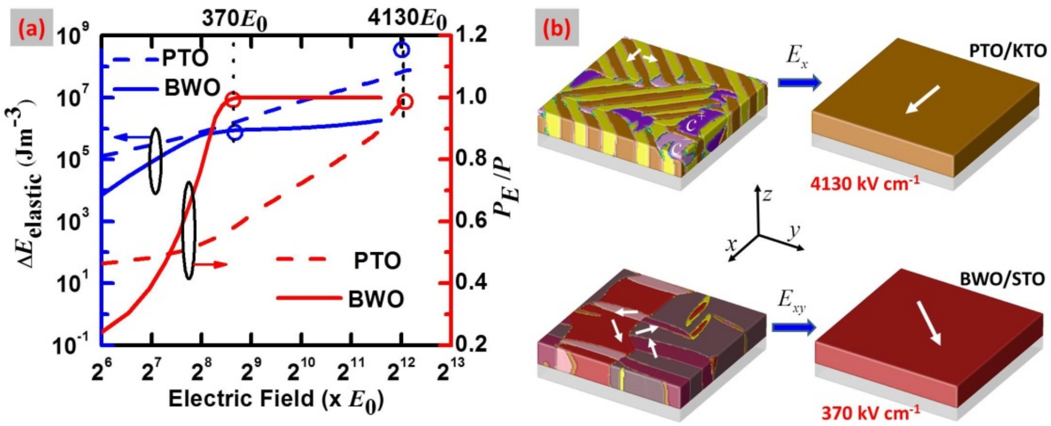

Supplementary Figure 8 | Simulation of in-plane elastic switching of BWO and PTO using planar field. (a) Variations of switching areal ratio (P_E/P) and elastic energy as a function of applied planer electric field for PTO and BWO thin films epitaxially grown on KTO and STO substrates, respectively. **(b)** Domain structures of PTO (upper panel) and BWO (below panel) before and after the fully ferroelastic switching. In **(a)**, E_0 represents 1 kVcm^{-1} and P_E indicates the polarization projection along the applied electric field direction. For PTO on KTO with tetragonal twin ferroelastic domain structures, the polarization is fully in the film plane and the electric field is applied along *x* direction, but for BWO with orthorhombic structure in the primitive cell, the electric field is applied along *xy* direction.

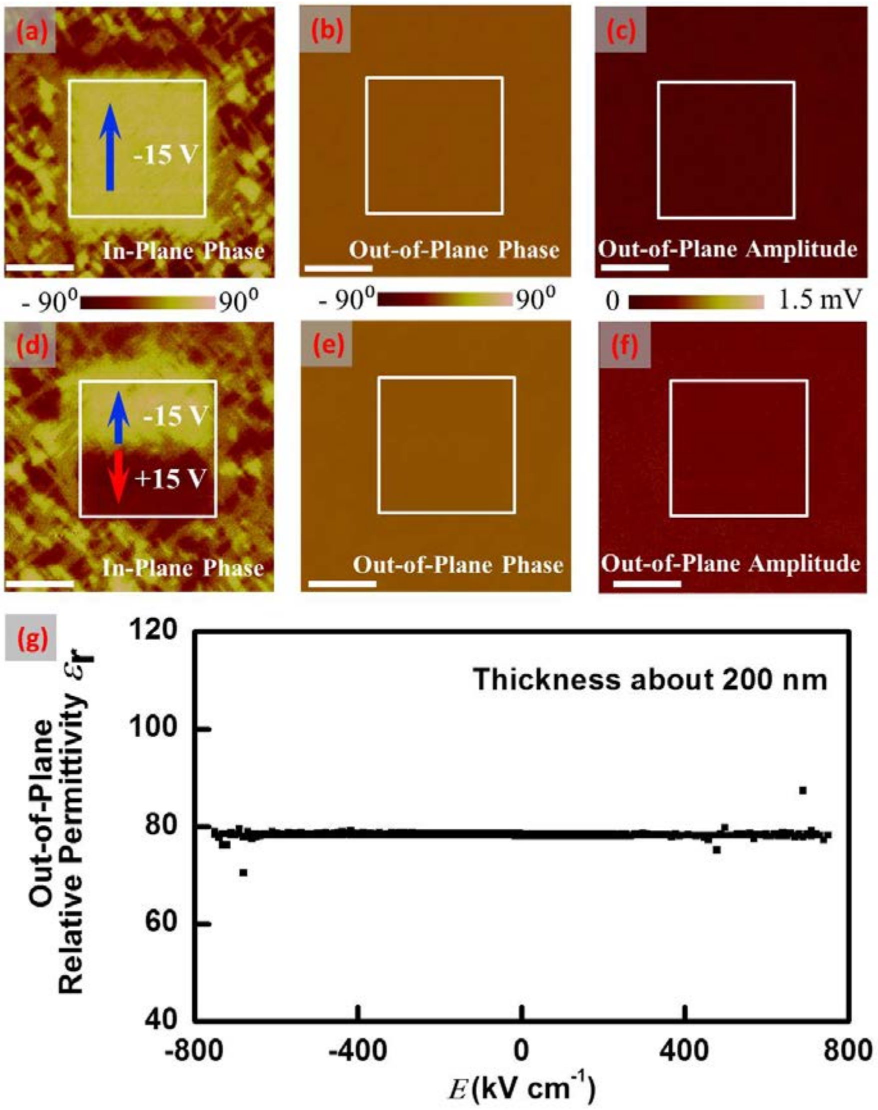

Supplementary Figure 9 | Zero out-of-plane polarization contribution in BWO films. **(a)** The in-plane phase image of the BWO thin film (thickness of ~60 nm) on STO substrate with SRO bottom electrode and a mono-domain was switched in the red box with probe bias of -15 V. **(b)** The corresponding out-of-plane phase image of the switched BWO thin film. **(c)** The corresponding out-of-plane amplitude image of the switched BWO thin film. **(d)** The in-plane phase image of the BWO thin film (thickness of ~60 nm) on STO substrate with SRO bottom electrode and two domains were switched in the red box with probe bias of ±15 V, respectively. **(e)** The corresponding out-of-plane phase image of the switched BWO thin film. **(f)** The corresponding out-of-plane amplitude image of the switched BWO thin film. The out-of-plane phase and amplitude images of the switched BWO domains indicate that

there is no any out-of-plane component of polarization signal above the noise level. Sacle bar, 1 μm **(a-f)**. **(g)** The out-of-plane relative permittivity of a 200 nm thick BWO film with SRO bottom electrode grown on STO substrate under the applied electric field from -800 $kVcm^{-1}$ to 800 $kVcm^{-1}$ shows that there is no out-of-plane polarization projection in the BWO film.

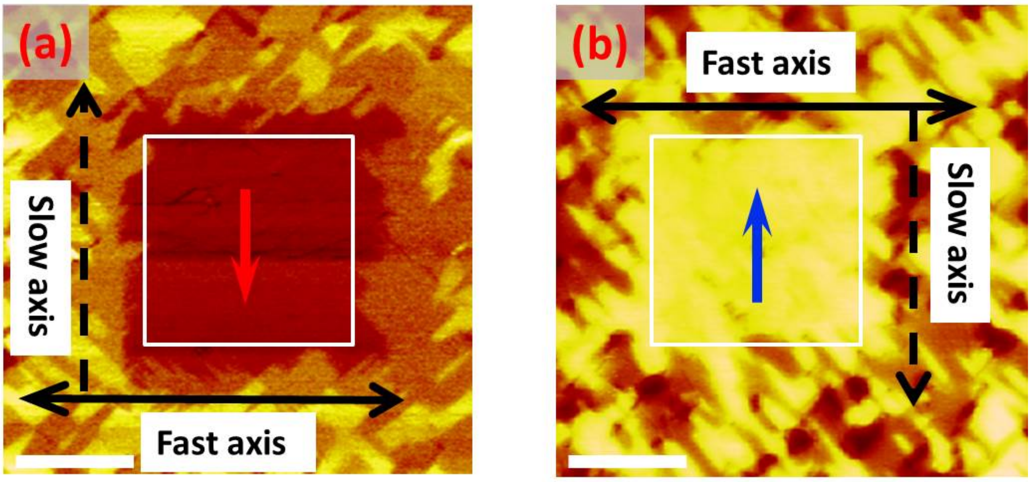

Supplementary Figure 10 | Nanoscale manipulation of ferroelastic mono-domain switching. PFM images after the application of a positive tip bias (+10 V) at nanoscale in BWO film grown on STO substrate with SRO bottom electrode show that in-plane domain switching is dependent on the slow-scanning directions of the cantilever. **(a)** The ferroelectric polarization (red arrow) is anti-parallel to the slow-scanning direction (downward). **(b)** A reversed slow-scanning direction (upward) also induces a reversed ferroelectric polarization (blue arrow). Sacle bar, 1 μm **(a-b)**.

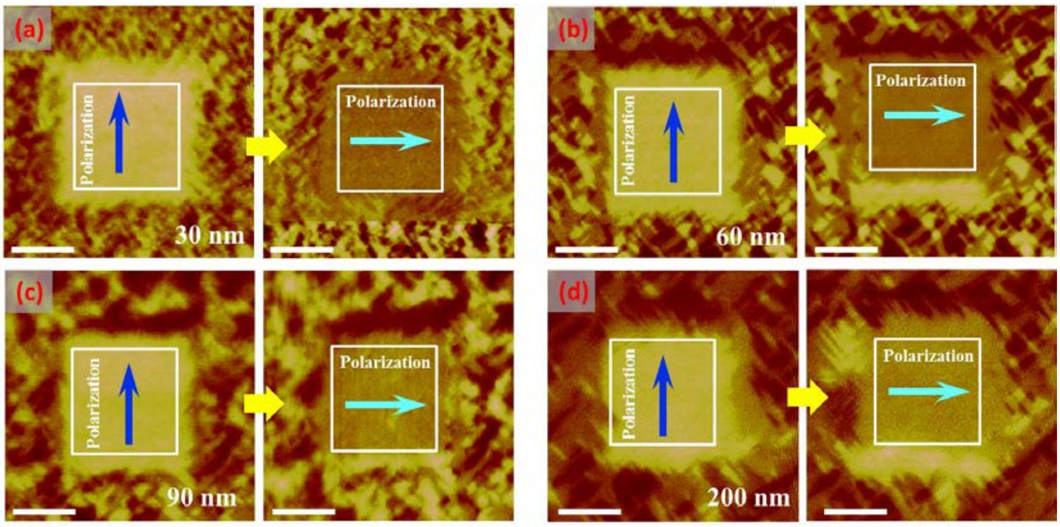

Supplementary Figure 11 | Thickness-dependent ferroelastic switching in BWO thin films. The PFM images of ferroelastic mono-domain switching in the **(a)** 30 nm, **(b)** 60 nm, **(c)** 90 nm and **(d)** 200 nm thick BWO films grown on (001) STO substrates with SRO bottom electrodes respectively. This thickness-dependent elastic switching help further understand the effect of substrate constraint has been strongly reduced in this orthorhombic BWO thin film. Sacle bar, 1 μm **(a-d)**.

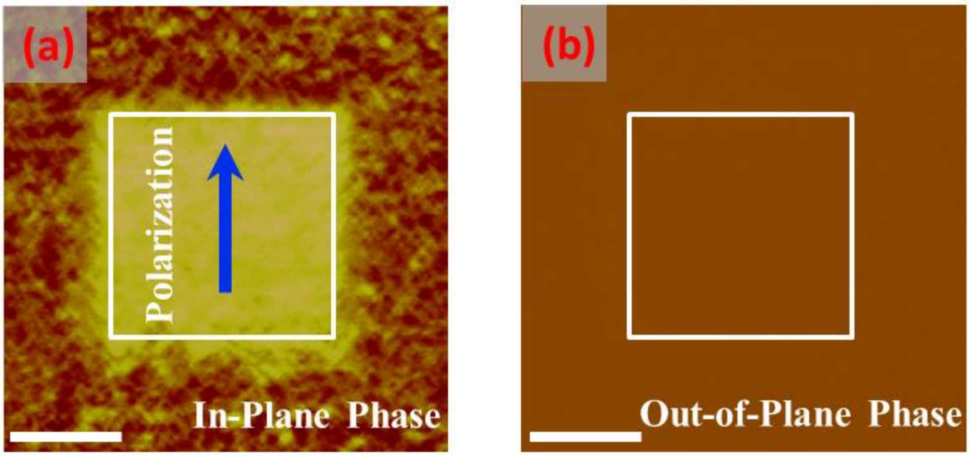

Supplementary Figure 12 | Ferroelastic domain switching of BWO film on (001) LAO substrate. The PFM image (in-plane **(a)** and out-of-plane **(b)** phase data) of the domain configuration after ferroelastic switching of BWO film on (001) LAO substrate with $La_{0.7}Sr_{0.3}MnO_3$ (LSMO) bottom electrode. The in-plane polarization is depicted by the blue arrow. This presents similar result as the ones presented in previous BWO on STO substrate (control of the switching of in-plane polarization). Sacle bar, 1 μm **(a-b)**.

Supplementary Note 1:

Phase-field simulation.

In phase-field method, the local polarization vector $\mathbf{P}=\left(P_1,P_2,P_3\right)$ is selected as the order parameter and its spatial distributions describe the ferroelectric domain structures. The time-dependent Landau-Ginzburg (TDGL) equation is employed to evolve the ferroelectric domain structures, i.e.:

$$\frac{\partial P_i\left(\mathbf{r},t\right)}{\partial t}=-L\frac{\delta F}{\delta P_i\left(\mathbf{r},t\right)} \tag{1}$$

with the kinetic coefficient (L) and the total free energy (F), respectively. Formulation of F can be expressed as:

$$F=\iiint_V\left(f_{\mathrm{bulk}}+f_{\mathrm{elastic}}+f_{\mathrm{electric}}+f_{\mathrm{grad}}\right)dV \tag{2}$$

with f_{bulk}, f_{elastic}, f_{electric}, and f_{grad} representing the densities of the bulk free energy, elastic energy, electrostatic energy, and gradient energy, respectively. For 001-oriented BWO, only in-plane spontaneous polarization emerges determined by the orthorhombic structure, thus the bulk free energy density can be expressed by a fourth-order Landau potential as following:

$$f_{\mathrm{bulk}}=\alpha_1\left(P_1^2+P_2^2\right)+\alpha_{11}\left(P_1^4+P_2^4\right)+\alpha_{12}P_1^2P_2^2\ . \tag{3}$$

Here α_1, α_{11}, and α_{12} are fitted using the method before [2], and $\alpha_1=4.03\times10^5\left(T-1200\right)\ \mathrm{V\ m\ C^{-1}}$, $\alpha_{11}=9.7\times10^8\ \mathrm{V\ m^5\ C^{-3}}$, and $\alpha_{12}=1.8\times10^9\ \mathrm{V\ m^5\ C^{-3}}$ determined from the experimental parameters [3].

The mathematical expressions for the f_{elastic}, f_{electric}, and f_{grad} are the same as before and can be found elsewhere [4,5]. In order to obtain the elastic and electrical energy densities, the mechanical equilibrium equation and electrostatic equilibrium equation need to be solved by considering the appropriate boundary conditions according to the status for the material to be, for example, bulk material [6], epitaxial

thin film [7], epitaxial nanowire [8], and free-standing nanoparticles [9]. In this work, domain switching in the single crystals and thin films for BWO and PTO without any pinning or defect are simulated for comparison. Material constants for BWO used in the simulation including the elastic constants, electrostrictive coefficients, dielectric constants, and gradient coefficients are [3,10]: $c_{11} = 1.22\times10^{11}\ \mathrm{N\ m^{-2}}$, $c_{12} = 0.14\times10^{11}\ \mathrm{N\ m^{-2}}$, $c_{44} = 0.54\times10^{11}\ \mathrm{N\ m^{-2}}$, $Q_{11} = 0.017\ \mathrm{C^{-2}\ m^{4}}$, $Q_{12} = -0.01\ \mathrm{C^{-2}\ m^{4}}$, $Q_{44} = 0.058\ \mathrm{C^{-2}\ m^{4}}$, $\kappa_{11} = \kappa_{22} = \kappa_{33} = 500$, $g_{11} = 2g_{44} = 6.0$, $g_{12} = 0.0$. The discrete grid points of $128\Delta x\times128\Delta y\times36\Delta z$ with real grid space Δx=Δy=10 nm, and Δz=2 nm, and the time step Δt=0.05 are employed to numerically solve the TDGL equation for BWO. The complete set of material constants for PTO can be found elsewhere [9].

Supplementary Note 2:

Control of in-plane mono-domain switching.

The electric field between two in-plane electrodes was controlled using an electric field of up to ~200 $kVcm^{-1}$ in samples with thickness ranging from 30 to 200 nm. The distribution of the electric field near the planar electrodes was inhomogeneous but in the center area between the electrodes the distribution of the electric field was uniform as a large aspect ratio (lateral length/ thickness of thin film) of the planar geometry. During the nanoscale control of domain switching using a scanning probe, the poling voltage on the electrically biased tip (contact radius approximately 5 nm) was controlled up to ±10 V_{dc}. The writing rate was 2 μms^{-1}. The in-plane distribution of the electric field is obtained based on the following equation [11]:

$$E = \frac{\mathrm{R}^2 V}{2\varepsilon_{\mathrm{r}}(\frac{t}{\varepsilon_{\mathrm{r}}}+R)r^2} \qquad (4)$$

where R, V, t and ε_r are the effective radius of the probe (approximately 120 nm), the applied tip bias (10 V), the thickness (approximately 200 nm) and the relative dielectric constant (approximately 70) of the film [10] respectively. The distribution distance (r) of electric field under the probe is considered from 5 nm to 100 nm.

Supplementary References:

1. Roytburd, A. L. Equilibrium structure of epitaxial layers. *Physica Status Solidi A* **37**, 329-339 (1976).

2. Wang, J. J., Wu, P. P., Ma, X. Q. and Chen, L. Q. Temperature-pressure phase diagram and ferroelectric properties of $BaTiO_3$ single crystal based on a modified Landau potential. *Journal of Applied Physics* **108**, 114105 (2010).

3. Zeng, T., Yan, H. X., Ning, H. P., Zeng, J. T. and Reece, M. J. Piezoelectric and ferroelectric properties of bismuth tungstate ceramics fabricated by spark plasma sintering. *Journal of the American Ceramic Society* **92**, 3108-3110 (2009).

4. Li, Y. L., Hu, S. Y., Liu, Z. K. and Chen, L. Q. Phase-field model of domain structures in ferroelectric thin films. *Applied Physics Letters* **78**, 3878-3880 (2001).

5. Wang, J. J. *et al*. Effect of strain on voltage-controlled magnetism in $BiFeO_3$-based heterostructures. *Scientific Reports* **4**, 4553 (2014).

6. Wang, J., Shi, S. Q., Chen, L. Q., Li, Y. and Zhang, T. Y. Phase field simulations of ferroelectric/ferroelastic polarization switching. *Acta Materialia* **52**, 749-764 (2004).

7. Li, Y. L., Hu, S. Y., Liu, Z. K. and Chen L. Q. Effect of substrate constraint on the stability and evolution of ferroelectric domain structures in thin films. *Acta Materialia* **50**, 395-411 (2002).

8. Wang, J. J. *et al*. Strain effect on phase transitions of $BaTiO_3$ nanowires. *Acta Materialia* **59**, 7189-7198 (2011).

9. Wang, J. J., Ma, X. Q., Li, Q., Britson, J. and Chen, L. Q. Phase transitions and domain structures of ferroelectric nanoparticles: Phase field model incorporating strong elastic and dielectric inhomogeneity. *Acta Materialia* **61**, 7591-7603 (2013).

10. Takeda, H. et al. Growth and piezoelectric properties of ferroelectric Bi_2WO_6 mono-domain crystals. *Solid State Communication* **150**, 836-839 (2010).

11. Durkan, C., Welland, M. E., Chu, D. P. and Migliorato, P. Probing domains at the nanometer scale in piezoelectric thin films. *Physical Review B* **60**, 16198-16204 (1999).
